# Palliative Radiation for Advanced Central Lung Tumors With Intentional Avoidance of the Esophagus (PROACTIVE): A Phase 3 Randomized Clinical Trial

**DOI:** 10.1001/jamaoncol.2021.7664

**Published:** 2022-02-24

**Authors:** Alexander V. Louie, Patrick V. Granton, Alysa Fairchild, Andrea Bezjak, Darin Gopaul, Liam Mulroy, Anthony Brade, Andrew Warner, Brock Debenham, David Bowes, Joda Kuk, Alexander Sun, Douglas Hoover, George B. Rodrigues, David A. Palma

**Affiliations:** 1 Department of Oncology, Western University, London Health Sciences Centre, London, Ontario, Canada; 2 Department of Radiation Oncology, University of Toronto, Sunnybrook Health Sciences Centre, Toronto, Ontario, Canada; 3 Department of Radiotherapy, Erasmus Medical Center, Rotterdam, the Netherlands; 4 Department of Oncology, University of Alberta, Edmonton, Alberta, Canada; 5 Department of Radiation Oncology, University of Toronto, Princess Margaret Cancer Centre, Toronto, Ontario, Canada; 6 Grand River Regional Cancer Centre, Kitchener, Ontario, Canada; 7 Department of Radiation Oncology, Dalhousie University, Halifax, Nova Scotia, Canada; 8 Department of Radiation Oncology, University of Toronto, Credit Valley Cancer Centre, Mississauga, Ontario, Canada

## Abstract

**Importance:**

Palliative thoracic radiotherapy (RT) can alleviate local symptoms associated with advanced non–small cell lung cancer (NSCLC), but esophagitis is a common treatment-related adverse event. Whether esophageal-sparing intensity-modulated RT (ES-IMRT) achieves a clinically relevant reduction in esophageal symptoms remains unclear.

**Objective:**

To examine whether ES-IMRT achieves a clinically relevant reduction in esophageal symptoms compared with standard RT.

**Design, Setting, and Participants:**

Palliative Radiation for Advanced Central Lung Tumors With Intentional Avoidance of the Esophagus (PROACTIVE) is a multicenter phase 3 randomized clinical trial that enrolled patients between June 24, 2016, and March 6, 2019. Data analysis was conducted from January 23, 2020, to October 22, 2021. Patients had up to 1 year of follow-up. Ninety patients at 6 tertiary academic cancer centers who had stage III/IV NSCLC and were eligible for palliative thoracic RT (20 Gy in 5 fractions or 30 Gy in 10 fractions) were included.

**Interventions:**

Patients were randomized (1:1) to standard RT (control arm) or ES-IMRT. Target coverage was compromised to ensure the maximum esophagus dose was no more than 80% of the RT prescription dose.

**Main Outcomes and Measures:**

The primary outcome was esophageal quality of life (QOL) 2 weeks post-RT, measured by the esophageal cancer subscale (ECS) of the Functional Assessment of Cancer Therapy: Esophagus questionnaire. Higher esophageal cancer subscale scores correspond with improved QOL, with a 2- to 3-point change considered clinically meaningful. Secondary outcomes included overall survival, toxic events, and other QOL metrics. Intention-to-treat analysis was used.

**Results:**

Between June 24, 2016, and March 6, 2019, 90 patients were randomized to standard RT or ES-IMRT (median age at randomization, 72.0 years [IQR, 65.6-80.3]; 50 [56%] were female). Thirty-six patients (40%) received 20 Gy and 54 (60%) received 30 Gy. For the primary end point, the mean (SD) 2-week ECS score was 50.5 (10.2) in the control arm (95% CI, 47.2-53.8) and 54.3 (7.6) in the ES-IMRT arm (95% CI, 51.9-56.7) (*P* = .06). Symptomatic RT-associated esophagitis occurred in 24% (n = 11) of patients in the control arm vs 2% (n = 1) in the ES-IMRT arm (*P* = .002). In a post hoc subgroup analysis based on the stratification factor, reduction in esophagitis was most evident in patients receiving 30 Gy (30% [n = 8] vs 0%; *P* = .004). Overall survival was similar with standard RT (median, 8.6; 95% CI, 5.7-15.6 months) and ES-IMRT (median, 8.7; 95% CI, 5.1-10.2 months) (*P* = .62).

**Conclusions and Relevance:**

In this phase 3 randomized clinical trial, ES-IMRT did not significantly improve esophageal QOL but significantly reduced the incidence of symptomatic esophagitis. Because post hoc analysis found that reduced esophagitis was most evident in patients receiving 30 Gy of RT, these findings suggest that ES-IMRT may be most beneficial when the prescription dose is higher (30 Gy).

**Trial Registration:**

ClinicalTrials.gov Identifier: NCT02752126

## Key Points

**Question:**

Can modern radiation techniques reduce the risk of radiation-associated esophageal adverse effects in patients with advanced lung cancer?

**Findings:**

In this phase 3 randomized clinical trial of esophageal-sparing intensity-modulated radiotherapy (ES-IMRT) or standard palliative radiotherapy for 90 patients with stage III/IV incurable non–small cell lung cancer, ES-IMRT significantly reduced symptomatic esophagitis (24% [n = 11] vs 2% [n = 1]), but did not significantly improve esophageal-related quality of life.

**Meaning:**

In this trial, the use of ES-IMRT did not definitively improve esophageal quality of life but reduced symptomatic esophagitis in patients with advanced lung cancer who were receiving palliative thoracic radiotherapy; this technique holds merit for translation into clinical practice.

## Teaser

This randomized clinical trial compares the use of esophageal-sparing intensity-modulated radiotherapy with standard palliative radiotherapy in patients with incurable non–small cell lung cancer.

## Introduction

**Figure 1.**
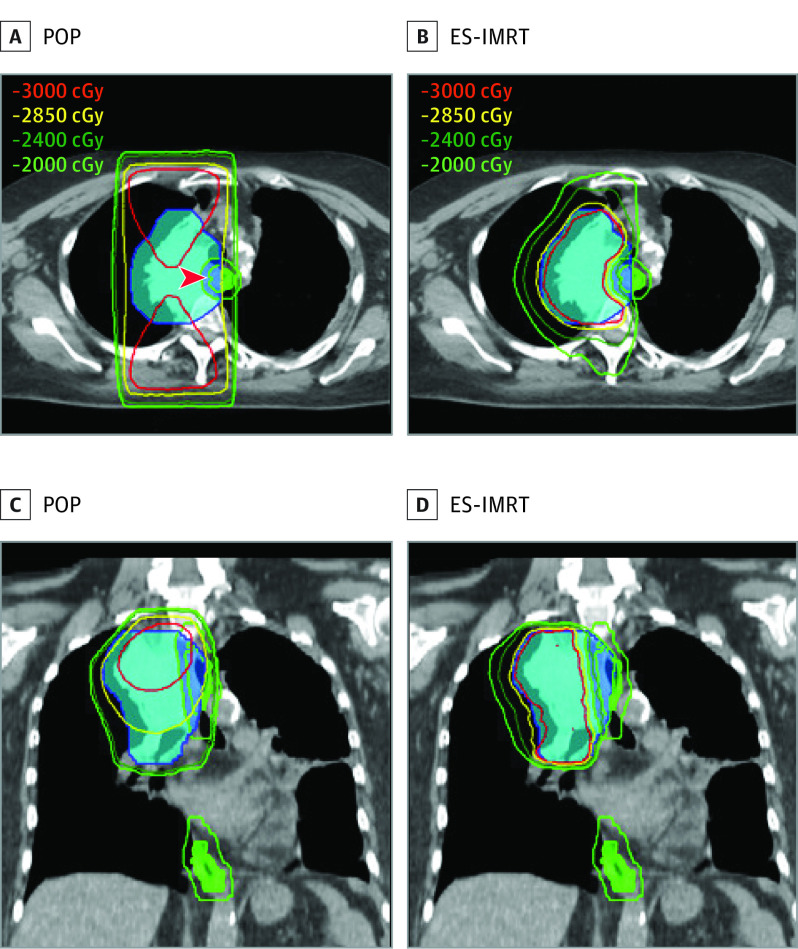
Depiction of Standard Radiation, Delivered as a Parallel-Opposed Pair (POP) in the Control Arm and Esophageal-Sparing Intensity-Modulated Radiotherapy (ES-IMRT) in the Experimental Arm The target is shown in blue color wash, the esophagus in green color wash, with the scale for dose lines shown at top left. Note the bowing-in of the higher isodose lines away from the esophagus in the ES-IMRT panel. (Figure from Granton et al^2^; reproduced under Creative Commons CC-BY license.)

Patients with advanced non–small cell lung cancer (NSCLC) are routinely treated with palliative thoracic radiotherapy (RT) to relieve tumor-related symptoms.^1^ In this setting, when cure is not possible, the central paradigm is to prolong survival and improve quality of life (QOL) while minimizing treatment burden and adverse effects. Palliative thoracic RT is traditionally delivered using simple anterior and posterior beams, called a parallel-opposed pair (POP) (Figure 1^2^), a technique that does not allow for specific avoidance of the esophagus in most cases. Although higher palliative doses of RT, such as 30 Gy in 10 fractions, are associated with a modest improvement in overall survival (OS) compared with lower doses, this level of dosing is at the cost of an increased risk of esophagitis and dysphagia,^3^ which can sometimes result in severe pain, weight loss, and occasionally hospitalization.^4^ A Cochrane review of randomized clinical trials comparing different doses for palliation of NSCLC reported a grade 3 or higher esophagitis rate of 25.7% with such dose regimens and 22.3% in patients receiving lower doses, such as 20 Gy in 5 fractions.^5^

Modern RT techniques may be able to mitigate this rate of esophagitis.^2^ Intensity-modulated radiotherapy (IMRT) is a technique that delivers an RT dose distribution that is highly conformal to the tumor target, allowing for optimal healthy tissue sparing.^6^ Although IMRT is more resource intensive and costly, its dosimetric and clinical benefits with high-dose, curative RT doses in locally advanced NSCLC are well documented.^7^ In contrast, there are limited data prospectively evaluating the potential utility of IMRT when using palliative RT doses.^8^ A prior modeling study suggested that the use of esophageal-sparing (ES)-IMRT when treating patients with palliative thoracic RT would reduce the rate of symptomatic esophagitis to 2%.^2^

Because patients with incurable NSCLC have a limited life expectancy, mitigating the QOL detriment from any palliative treatments is paramount. We therefore conducted this phase 3 randomized clinical trial to measure the effect of ES-IMRT on treatment-related esophageal toxic effects in incurable NSCLC.

## Methods

### Study Design

The Palliative Radiation for Advanced Central Lung Tumors With Intentional Avoidance of the Esophagus (PROACTIVE) study was an investigator-initiated, multi-institutional, open-label, parallel-group, phase 3 randomized clinical trial conducted by the Canadian Pulmonary Radiotherapy Investigators group at 6 Canadian academic centers. Patients with biopsy-proven stage III/IV NSCLC who were being considered for palliative RT were stratified by intended-dose prescription (20 Gy in 5 fractions vs 30 Gy in 10 fractions), with the selection of RT dose at the discretion of the treating radiation oncologist. Patients were randomly assigned in a 1:1 ratio to receive either standard palliative thoracic RT (control arm; RT delivered using conventional radiation without esophageal sparing, with a POP technique preferred) or ES-IMRT. Appropriate regulatory and ethics approval was obtained in all jurisdictions and this study was approved by the Western University Research Ethics Board. All patients provided written informed consent; participants did not receive financial compensation. The full protocol is provided in Supplement 1. This study followed the Consolidated Standards of Reporting Trials (CONSORT) reporting guideline.

### Participants

**Figure 2.**
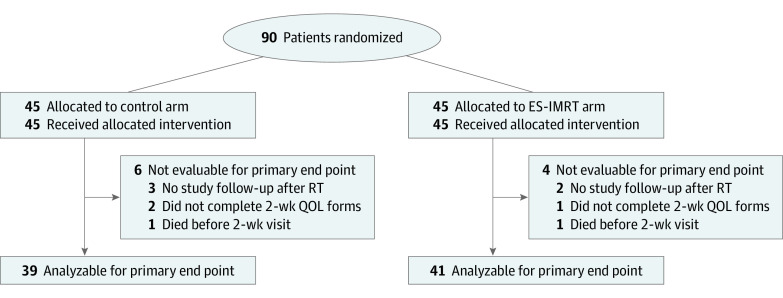
Consolidated Standards of Reporting Trials Diagram ES-IMRT indicates esophageal-sparing intensity-modulated radiotherapy; QOL, quality of life; and RT, radiotherapy.

Patients were required to be aged 18 years or older, with Eastern Cooperative Oncology Group performance status 0 to 3, a life expectancy of 3 or more months, and advanced NSCLC, defined as American Joint Committee on Cancer 7th edition stage III/IV, not eligible for curative-intent treatment. Concurrent palliative RT to metastatic sites other than the stomach and/or liver was permissible because RT to those sites could cause symptoms that affect the primary end point independent of randomization arm. Prior planned systemic treatment was allowed provided that none was given within 2 weeks before RT, concurrent with RT, or within a 2-week period post-RT. Patients were required to have at least 5 cm of the esophagus in the intended standard, non-IMRT treatment plan (Figure 2).

The main exclusion criteria included prior thoracic RT, pregnancy/lactation, serious medical comorbidities that prohibited RT, severe disorders of the esophagus, and inability to attend the full course of RT or planned follow-up. Radiotherapy with field overlap with the stomach was also not allowed, owing to its potential effect on the end points of interest.

### Randomization

Patients were assigned to groups using a computer-generated randomization list with permuted blocks of 6 (with the block size known only to the statistician until completion of the study), stratified by the intended dose (20 Gy in 5 fractions vs 30 Gy in 10 fractions). Patients were allocated after receipt of a completed enrollment form and other regulatory documents. These documents were faxed to the coordinating center and treatment allocation was communicated by email. Neither patients nor enrolling physicians were blinded to treatment allocation.

### Procedures

All patients underwent 4-dimensional computed tomography simulation with 3-mm thick contiguous slices for RT planning. Simulation was in the treatment position, with arms placed above the head, if possible. Target and organ at risk contours were delineated before randomization per International Commission on Radiation Units definitions.

The minimum length of the esophagus in the field (defined as the length of esophagus receiving >50% of the prescription dose at any point along its circumference) was mandated to be at least 5 cm for enrollment. Prior to randomization, the standard RT plan was created and uploaded to a central office to confirm that 5 cm of esophagus would be within the treatment field and avoid any changes of the standard plan after randomization. If the patient was randomized to the control arm, the standard RT plan was delivered as designed. If randomized to ES-IMRT, then an IMRT plan was created. Full RT planning details are available in the eAppendix in Supplement 2. If the radiation target (ie, the planning tumor volume) and the esophagus were within close proximity, the plan was designed such that the esophageal constraint took priority in the RT planning optimization process; in some instances, the tumor target was compromised to achieve esophageal planning constraints. Before trial activation, each participating site completed 2 test planning cases to ensure protocol adherence.

### Outcomes

The primary study end point was esophageal QOL at 2 weeks following RT, as measured by the esophageal cancer subscale (ECS) of the Functional Assessment of Cancer Therapy: Esophagus (FACT-E) questionnaire. Higher ECS scores correspond with improved QOL, with a 2- to 3-point change in the total score considered clinically significant. Secondary end points included toxic events (scored using Common Terminology Criteria for Adverse Events, version 4.0 by a nurse asking the patient about their symptoms using a standardized rubric), pulmonary QOL, general QOL, and OS, defined as time from randomization to death from any cause. The additional secondary end points of cost-effectiveness and dosimetric comparison of plans will be reported as future analyses.

Patients completed Functional Assessment of Cancer Therapy: General (FACT-G), FACT-E, Functional Assessment of Cancer Therapy: Lung (FACT-L) and EuroQOL 5-Dimension 5-Level (EQ-5D-5L) questionnaires at baseline and at each scheduled follow-up, which were at 2 weeks, 4 weeks, 8 weeks, 3 months, 6 months, and 1-year post-RT. At the same surveillance time points, adverse events were evaluated using Common Terminology Criteria for Adverse Events, version 4.0. Additional surveillance, laboratory, and imaging investigations were at the discretion of the treating oncologist.

### Statistical Analysis

Data analysis was conducted from January 23, 2020, to October 22, 2021. Regarding the primary end point, a moderately large effect size (Cohen *d*, 0.65), and a nonresponder rate of 15% to QOL questionnaires were assumed.^9^ With that effect size, and using a 2-sided, independent-sample *t* test with an α level of .05 and power of 80%, 90 patients (45 in each arm) were required.

All patients were analyzed using the intention-to-treat principle, and all analyses were prespecified, unless otherwise indicated below. Overall survival was estimated using the Kaplan-Meier method, with differences compared using the stratified log-rank test. Differences in rates of grade 2 or higher toxic effects between treatment arms were compared using the χ^2^ test or Fisher exact test as appropriate. Multivariable logistic regression analysis was performed to identify predictors of grade 2 or higher esophagitis. All eligible variables with univariable *P* values <.05 were incorporated into a multivariable regression model and sequentially removed using backward elimination techniques until all remaining covariates had *P* values <.05. Quality of life was measured using FACT-G, FACT-E, FACT-L, and EQ-5D-5L, with differences at 2 weeks following RT compared using the independent-sample *t* test. Quality of life differences over time between groups were compared using linear mixed modeling (with time, treatment arm, interaction between treatment arm and time, and intended dose as fixed effects and patient number as a random effect). All linear mixed models were generated based on data ordered by patient and follow-up visit and using unstructured covariance matrices and maximum likelihood estimation. All models included intercepts and no residual variance adjustments were applied.

One interim analysis was conducted after 30 patients were accrued. The protocol specified that the data safety and monitoring committee would recommend stopping the trial if there was a statistically significant difference of *P* < .001 using the independent-sample *t* test for ECS score or the stratified log-rank test (stratified by intended dose) for OS (Haybrittle-Peto stopping rule) between the 2 arms. These criteria were not met, and the trial proceeded to full accrual, which was completed in March 2019.

All statistical analyses were performed using SAS, version 9.4 software (SAS Institute Inc), using 2-sided statistical testing at the .05 significance level. Statistical methods and results for sensitivity analyses are provided in the eMethods and eResults in Supplement 2.

## Results

### Baseline Characteristics

Between June 24, 2016, and March 6, 2019, 90 patients were randomized at 6 Canadian centers (Figure 2). Baseline characteristics are reported in Table 1. Most patients had stage IV (64 [71%]) NSCLC and good (Eastern Cooperative Oncology Group 0-1) performance status (65 [72%]). The most common histologic type was adenocarcinoma (43 [48%]). Thirty-six patients (40%) received 20 Gy in 5 fractions and 54 (60%) received 30 Gy in 10 fractions. In the standard RT arm, the mean (SD) esophagus dose was 10.2 (3.9) Gy and the maximum (SD) esophagus dose was 25.3 (5.9) Gy. In the ES-IMRT arm, the mean (SD) esophagus dose was 9.8 (5.4) Gy and the maximum (SD) esophagus dose was 23.8 (5.9) Gy. The maximum spinal cord dose was 25.3 (7.3) Gy in the standard RT arm and 22.0 (9.0) Gy in the ES-IMRT arm. Planning tumor volume D95 was 24.5 (4.8) Gy in the standard RT arm and 23.8 (4.5) Gy in the ES-IMRT arm. Eleven patients (12%) experienced treatment delays (7 in standard RT arm and 4 in ES-IMRT arm). In total, 22 of 45 patients in the ES-IMRT arm had cancer (gross tumor volume) in direct contact with the esophagus and would have had, at most, a 20% reduction in dose at the area touching the esophagus. The mean (SD) number of cycles of further systemic therapy was 7.7 (7.1) in the standard arm and 5.1 (3.2) in the ES-IMRT arm (*P* = .68).

**Table 1.** Baseline Characteristics

Characteristic	No. (%)			
	No.	All patients (N = 90)	Control arm (n = 45)	ES-IMRT arm (n = 45)
Age at randomization, median (IQR), y	90	72.0 (65.6-80.3)	72.1 (65.6-77.9)	71.9 (67.9-81.8)
Sex	90			
Female		50 (56)	24 (53)	26 (58)
Male		40 (44)	21 (47)	19 (42)
Intended dose	90			
20 Gy in 5 fractions		36 (40)	18 (40)	18 (40)
30 Gy in 10 fractions		54 (60)	27 (60)	27 (60)
Planned esophagus length, mean (SD), cm	90	10.1 (3.4)	10.5 (3.0)	9.8 (3.8)
Previous treatment^a^				
Any type	90	14 (16)	7 (16)	7 (16)
Radiotherapy	90	9 (10)	5 (11)	4 (9)
Surgery	90	3 (3)	2 (4)	1 (2)
Chemotherapy	90	9 (10)	5 (11)	4 (9)
Histologic findings	89			
Adenocarcinoma		43 (48)	19 (43)	24 (53)
Squamous		24 (27)	13 (29)	11 (24)
NSCLC NOS		22 (25)	12 (27)	10 (22)
Stage	90			
III		26 (29)	15 (33)	11 (24)
IV		64 (71)	30 (67)	34 (76)
ECOG performance status	90			
0		12 (13)	5 (11)	7 (16)
1		53 (59)	25 (56)	28 (62)
2		20 (22)	12 (27)	8 (18)
3		5 (6)	3 (7)	2 (4)
Baseline weight, mean (SD), kg	88	71.4 (16.0)	69.7 (14.6)	73.2 (17.3)

Abbreviations: ECOG, Eastern Cooperative Oncology Group; ES-IMRT, esophageal-sparing intensity-modulated radiotherapy; NOS, not otherwise specified; NSCLC, non-small cell lung cancer.

^a^ Categories are not mutually exclusive as some patients received multiple previous treatments and 76 of 90 patients did not receive any previous treatment.

### Outcomes

Eighty patients completed the ECS QOL forms at 2 weeks following RT and were therefore analyzable for the primary end point. The mean (SD) 2-week ECS score was 50.5 (10.2) (95% CI, 47.2-53.8) in the standard RT arm and 54.3 (7.6) in the ES-IMRT arm (95% CI, 51.9-56.7) (*P* = .06), corresponding with an effect size of 3.8 (95% CI, −0.2 to 7.8). In a post hoc subgroup analysis dividing patients by the stratification factor, this benefit was mostly observed in patients receiving 30 Gy (51.1 [10.7] vs 56.4 [7.1]; *P* = .06), rather than in those receiving 20 Gy (49.7 [9.8] vs 50.9 [7.2]; *P* = .68). As a sensitivity analysis, 2-week ECS by arm was further examined by planned esophagus length, histologic characteristics, stage, and Eastern Cooperative Oncology Group performance status (eResults, eTable in Supplement 2). There were no significant differences between arms in ECS scores at other time points, and no differences between arms in other QOL metrics (FACT-G total, FACT-L, or EQ-5D-5L scores), either at 2 weeks following RT or over the entire course of follow-up. No significant interactions were observed between treatment arm and time. The QOL metrics over time are shown in the eFigure in Supplement 2.

The incidence of grade 2 or higher esophagitis was 24% (n = 11) in the standard RT arm vs 2% (n = 1) in the ES-IMRT arm (*P* = .002). Similarly, in a post hoc subgroup analysis dividing patients by the stratification factor, this benefit was mostly observed in patients receiving 30 Gy (8 [30%] vs 0; *P* = .004), rather than in those receiving 20 Gy (3 [17%] vs 1 [6%]; *P* = .60). On exploratory multivariable analysis, prior chemotherapy (odds ratio [OR], 9.33; 95% CI, 1.53-56.82; *P* = .02) was also predictive of symptomatic esophagitis, as was randomization to the control arm (OR, 16.83; 95% CI, 1.85-152.84; *P* = .01), stratified by intended dose. Intended dose of 30 Gy vs 20 Gy was not significantly predictive of symptomatic esophagitis (OR, 1.37; 95% CI, 0.38-5.00; *P* = .63), whereas randomization to the control arm was significantly predictive (OR, 9.74; 95% CI, 1.70-55.87; *P* = .01). A summary of all adverse events, categorized by grade and type and stratified by treatment arm is presented in Table 2, with no other differences between arms apart from esophagitis rates.

**Table 2.** Treatment-Related Adverse Events for All Patients Stratified by Treatment Arm

Adverse event	CTCAE, version 4, grade										*P* value^a^
	Control arm (n = 45)					ES-IMRT arm (n = 45)					
	1	2	3	4	5	1	2	3	4	5	
Anorexia	1	1	0	0	0	2	0	0	0	0	>.99
Cough	1	0	0	0	0	1	0	0	0	0	NA
Diarrhea	1	0	0	0	0	0	0	0	0	0	NA
Dyspnea	0	0	0	1	0	0	0	0	1	0	>.99
Esophagitis	3	11	0	0	0	6	1	0	0	0	.002
Fatigue	6	9	0	0	0	8	6	0	0	0	.40
Headache	0	0	0	0	0	1	0	0	0	0	NA
Nausea	1	0	0	0	0	2	0	0	0	0	NA
Odynophagia	1	0	0	0	0	0	0	0	0	0	NA
Pain	0	3	0	0	0	1	1	0	0	0	.62
Pneumonitis	1	2	0	0	0	0	0	2	0	0	>.99
Skin	5	1	0	0	0	0	0	0	0	0	>.99
Voice changes	1	0	0	0	0	0	0	0	0	0	NA
Weakness	0	1	0	0	0	0	0	0	0	0	>.99

Abbreviations: CTCAE, Common Terminology Criteria for Adverse Events; ES-IMRT, esophageal-sparing intensity-modulated radiotherapy; NA, not applicable (no grade ≥2 adverse events).

^a^ *P* values calculated based on rates of grade 2 or higher adverse events using χ^2^ test or Fisher exact test as appropriate.

**Figure 3.**
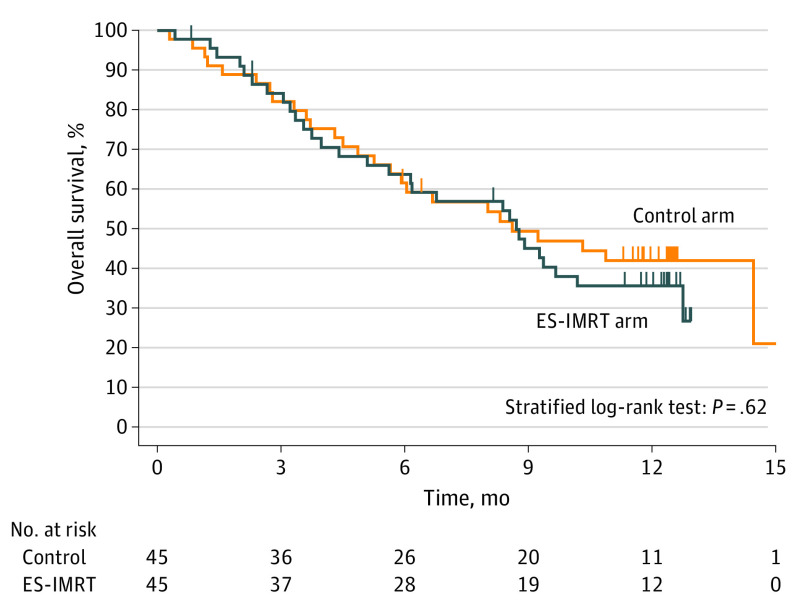
Overall Survival ES-IMRT indicates esophageal-sparing intensity-modulated radiotherapy.

There were 56 death events during the 1-year follow-up period, 27 in the control arm and 29 in the ES-IMRT arm. Overall survival was similar between arms, with a median OS of 8.6 (95% CI, 5.7-15.6) months in the control arm vs 8.7 (95% CI, 5.1-10.2) months in the ES-IMRT arm (stratified log-rank test: *P* = .62; Figure 3).

## Discussion

In this phase 3 randomized clinical trial, ES-IMRT for advanced NSCLC did not meet the primary end point of a 2-week improvement in FACT-E esophageal QOL compared with standard palliative RT, but showed nonsignificant improvement in the primary end point and achieved a substantial improvement in symptomatic esophagitis rates. The low rates of grade 2 esophagitis reported herein (2% in the ES-IMRT arm, with no instances of higher-grade esophagitis), compare favorably with previously reported 22% to 25% rate of grade 3 or higher esophagitis in the Cochrane review.^5^ Considering these findings in sum, ES-IMRT may hold value in reducing esophageal adverse effects compared with standard thoracic RT.

To our knowledge, our findings represent the first randomized evidence addressing whether ES-IMRT may hold value in reducing esophageal adverse effects and build on the growing literature indicating that advanced radiation techniques can reduce esophagitis in this population. The single-arm Cancer Trials Ireland 06-34 trial aimed to reduce esophagitis rates in patients receiving palliative RT for lung cancer.^10^ In that trial, a 3-dimensional conformal radiation technique that allowed multiple beam angles and blocks with the goal of sparing the esophagus was used. This approach is more advanced than the POP technique used in the standard arm of our trial and is an intermediate approach between the 2 arms of the PROACTIVE trial. There were no instances of grade 3 or higher esophagitis, the primary end point of the Cancer Trials Ireland 06-34, which is a significant finding compared with their prespecified expected historical rate of grade 3 or higher esophagitis of 35% (*P* < .0005).^10^ They reported low rates of grade 2 or higher esophagitis—11% during treatment and 7% 2 weeks posttreatment. Although other techniques can offer esophageal sparing, IMRT provides a distinct advantage in that it allows sculpting of the dose around the esophagus in a manner that other approaches cannot provide. In the setting of stage III NSCLC treated with concurrent chemoradiation, a retrospective study comparing 44 patients treated with esophageal sparing vs 43 treated with a conventional approach reported improvements in both grade 3 esophagitis (4.5% vs 30.2%; *P* = .002) and nutritional status (81.8% vs 58.1%; *P* = .045).^11^ A phase 1 trial of esophageal sparing in the setting of concurrent chemoradiation (70 Gy) for locally advanced NSCLC or small-cell lung cancer reported a grade 3 or higher esophagitis rate of 0%, a promising finding given the historically high rate of esophagitis in this population.^12^ Taken together, these studies provide data suggesting that reduction of dose to the esophagus is feasible and may result in clinically relevant gains.

### Strengths and Limitations

This study has strengths and limitations. The PROACTIVE trial is one of few randomized phase 3 studies in radiation oncology to evaluate patient-reported outcomes and QOL within a palliative patient population, and the completion rate of QOL metrics at 2 weeks surpassed the anticipated rate. However, changes in the ECS of the FACT-E may not have been sensitive enough to reflect the expected differences in symptomatic esophagitis, which may be the most clinically meaningful. Although this primary end point was not met, statistically significant reduction in the rate of symptomatic esophagitis with ES-IMRT is still a meaningful finding for patients in an end-of-life setting.

However, there are limitations to be borne in mind. The study was not blinded, and knowledge of treatment arm could conceivably affect patient responses. Nevertheless, blinding was not feasible, because the consent process included a description of the 2 arms and patients would be aware as to whether the machine was delivering 2 beams (in the POP arm) vs a more complex technique (in the ES-IMRT arm). In addition, we assumed a fairly large effect size (Cohen *d* = 0.65) in our power calculation, which may have led to underpowering to detect the true effect size, which was a Cohen *d* value of 0.42. The use of ES-IMRT has limitations compared with a POP technique. First, ES-IMRT takes more time to design and therefore may not be appropriate in the scenario in which more urgent thoracic RT is indicated. Technological advances, however, will allow ES-IMRT to be designed within 48 hours.^13^ Second, ES-IMRT is likely to be more costly, and cost-effectiveness will be the topic of a future study on the PROACTIVE cohort. Third, the ES-IMRT technique we describe herein represents a paradigm shift in palliative RT planning. Typically, the entire tumor target is treated with the full RT prescription doses in these scenarios; reduction of dose along the organ at risk and target interface is more commonly performed with curative and/or ablative doses. Therefore, there is still uncertainty as to whether using ES-IMRT produced equivalent results on the tumor, but that would require a much larger noninferiority study.

## Conclusions

In this phase 3 randomized clinical trial, ES-IMRT demonstrated no significant improvement in esophageal QOL but significantly reduced the incidence of symptomatic esophagitis. This benefit was most evident in patients receiving 30 Gy in 10 fractions. These findings suggest that ES-IMRT may be an option for patients in whom reduction of esophageal toxic events is important.

## Notes

Supplement 1. Trial Protocol

Supplement 2. **eAppendix.** Treatment Planning Guidelines for PROACTIVE **eAppendix.** Treatment Planning Guidelines for PROACTIVE eMethods. Sensitivity Analyses eResults. Sensitivity Analyses **eFigure.** Changes in Quality-of-Life Scores Over Time by Domain and by Treatment Arm **eTable.** Subgroup Analyses of FACT-E ECS 2 Weeks Postradiotherapy

Supplement 3. Data Sharing Statement
