# Supplementary material for: Palliative Radiation for Advanced Central Lung Tumors With Intentional Avoidance of the Esophagus (PROACTIVE): A Phase 3 Randomized Clinical Trial
Source: JAMA Oncol. 2022 Feb 24;8(4):1–7. doi: 10.1001/jamaoncol.2021.7664 (PMC8874872; doi:10.1001/jamaoncol.2021.7664)
Supplement: Supplement 1. — Trial Protocol [file jamaoncol-e217664-s001.pdf]

A randomized phase III study of **P**alliative **R**adiation **o**f **A**dvanced **C**entral lung **T**umors with **I**ntentional a**v**oidance of the **E**sophagus **(PROACTIVE)**

Principal Investigator

Alexander V. Louie, MD, PhD (Radiation Oncology)

Trial Steering Committee

David A. Palma, MD, PhD (Radiation Oncology)

Patrick Granton, PhD (Radiation Physics)

Devin Schellenberg, MD (Radiation Oncology)

Andrew Warner, MSc (Biostatistics)

STUDY SCHEMA

Biopsy-proven stage III/IV NSCLC, for palliative radiotherapy

↓

4DCT simulation (esophageal contrast optional)

↓

Stratify by Intended Dose Prescription
30 Gy / 10#
vs.
20 Gy / 5 #

↓

Randomize

Standard palliative radiation (e.g. POP) vs.
Esophageal sparing plan

↓

After RT, patients can receive palliative systemic therapy at the discretion of medical oncologist, but not within 2 weeks of last RT

↓

Follow-up at 2 weeks, 4 weeks, 8 weeks, 3 months, 6 months, 1 year post-RT

Primary Endpoint: esophageal quality of life (FACT-E) at 2 weeks following RT

Required Sample Size: 90 patients

Table of Contents

1.0 Introduction

2.0 Objectives

3.0 Trial Design

4.0 Study Population

5.0 Pre-Treatment Evaluation

6.0 Treatment Plan

7.0 Adverse Events

8.0 Subject Withdrawal

9.0 Follow-up Evaluation and Assessment of Efficacy

10.0 Statistical Considerations and Sample Size Calculation

11.0 Ethical Considerations

12.0 Authorship

13.0 Financial Support

APPENDICES

1. Eligibility Checklist

2. Follow-up schedule

3. Letter of Consent

4. Quality of Life Questionnaire

5. Common Toxicity Criteria

1.0 INTRODUCTION

1.1 Background

Palliative thoracic radiotherapy is an effective modality for symptom prevention and improvement in patients with metastatic non-small cell lung cancer. For a subset of these patients with good performance status (ECOG 0-1), a meta-analysis found that employing doses of 35 Gy_{10} and higher (e.g. 30 Gy in 10 fractions) through a standard conventional antero-posterior/postero-anterior (APPA) approach was associated with a modest survival benefit at 1-year, compared to lower doses. This however, was at the cost of an increased rate of esophagitis within 3 months (1). Esophagitis can lead to pain, weight loss, and hospital admissions, and overall can reduce quality of life. In some cases, specifically in the setting of stage III NSCLC, severe esophagitis after treatment has been associated with inferior overall survival (2), perhaps due to malnutrition.

In the modern era, there is wider availability of advanced radiotherapy techniques (e.g. CT-simulation, IMRT, arc-based therapy), allowing for increased conformality and sparing of organs at risk (OARs), allowing for conformal avoidance of organs at risk, such as the esophagus. Esophageal sparing has been employed for stage III NSCLC with early promising results (3,4), but to our knowledge has not been formally tested in the palliative setting. The goal of this study is to conduct a multi-institutional randomized phase III clinical trial comparing standard palliative treatment (APPA), using doses of 20 Gy in 5 fractions or 30 Gy in 10 fractions, versus the same dose using esophageal-sparing IMRT (ES-IMRT).

2.0 OBJECTIVES

To assess the impact of ES-IMRT versus standard palliative RT on quality of life, survival, toxicity and cost-effectiveness in patients with metastatic/locally advanced lung cancer, in the year after RT.

2.1 Endpoints

Primary Endpoint:

1. Esophageal quality of life
    - Measured by the Esophageal Cancer Subscale (ECS) of the FACT-E

Secondary Endpoints:

1. Pulmonary and General Quality of life
    - Pulmonary quality of life measured by the Lung Cancer Subscale (LCS) of the FACT-L
    - General quality of life measured by the FACT-G and the EQ-5D
2. Overall survival
    - Defined as time from randomization to death from any cause.
3. Toxicity
    - Assessed by the NCI-CTC v.4
4. Cost Effectiveness
5. Dosimetric comparisons of plans in both arms.

3.0 STUDY DESIGN

This study is designed as a randomized phase III study. Patients will be randomized between standard of care palliative thoracic RT vs. ES-IMRT.

Patients will be stratified by intended dose prescriptions of 30 Gy in 10 fractions or 20 Gy in 5 fractions, and the choice of dose is pre-specified before randomization.

Patients will be randomized in a 1:1 ratio to either:

Arm 1: Standard Radiotherapy (delivered as 3-D conformal radiotherapy without esophageal sparing). An APPA technique is preferred.

Arm 2: Esophageal Sparing Radiotherapy using IMRT. Patients will receive the same pre-specified dose, but with esophageal sparing.

In either arm, radiotherapy must begin no later than 2 weeks from the time of randomization, although treatment as early as possible is highly encouraged.

4.0 PATIENT SELECTION

4.1 Inclusion Criteria

1. AJCC 7th edition stage IV NSCLC or stage III not eligible for curative intent treatment
2. Intended to receive palliative radiotherapy to the thorax, to a dose of 30 Gy

in 10 fractions or 20 Gy in 5 fractions. In either treatment arm, at least **5 cm of the esophagus** should be in the intended treatment field.

3. Willingness and ability to provide informed consent
4. ECOG performance status 0-3
5. Age 18 years or older
6. Prior or planned systemic treatment (chemotherapy, immunotherapy, targeted agents) is permissible at the discretion of the treating medical oncologist, provided that no systemic treatment is given within 2 weeks prior to RT, concurrent with RT, or within a 2-week period post RT.
7. Concurrent palliative RT to other metastatic sites is permissible other than the stomach and/or liver
8. Life expectancy > 3 months

4.2 Exclusion Criteria

1. Prior thoracic RT
2. Serious medical comorbidities, which in the opinion of the radiation oncologist preclude the delivery of RT
3. Pregnant or lactating women
4. Inability to attend the full course of RT or planned follow up/survey responses.
5. Congenital abnormalities of the esophagus or severe disorders of the esophagus (e.g. achalasia)

5.0 PRE-TREATMENT EVALUATION

The following are mandatory:

i. History and physical examination, with documentation of weight and performance status (ECOG)
ii. CT chest within 3 months.
iii. Bone scan and brain imaging (CT or MRI) are recommended if there are signs or symptoms consistent with metastases at those sites
iv. Negative serum or urine pregnancy test for women of child-bearing age
v. Histological confirmation of NSCLC malignancy
vi. Informed consent

6.0 TREATMENT PLAN

In either treatment arm, concurrent chemotherapy, targeted therapy, and/or immunotherapy is not permitted within the 2 weeks prior to RT, during RT, or 2 weeks following RT. Permissible doses are 30 Gy in 10 fractions or 20 Gy in 5 fractions, and this must be specified prior to randomization.

6.01 Test Cases

Prior to trial activation, each participating site must complete two test planning cases to ensure protocol compliance. These cases will be provided in anonymized form by secure FTP transfer. For further details, please see section 6.4.

6.02 Immobilization and positioning

Treatment will be set up using reproducible positioning, verified using an on-line

protocol, for all patients in this study. Immobilization may include items such as a chest board, vac-lok bag and/or double leg immobilizer, as per individual institutional practice.

6.03 Radiation Simulation

For all patients, a 4D-CT will be performed with 3 mm thick contiguous CT slices and the scanned volume should encompass at least C5 to below the diaphragm. Simulation will be in the treatment position (e.g. arms placed above the head if possible). Oral contrast to aid in the delineation of the esophagus (i.e. esophacat) is optional at the time of simulation. IV contrast to improve the delineation of target volumes and organs at risk is optional.

6.04 Target Volumes and Organs at Risk (OAR)

Prior to randomization, all patients will have the following contours delineated on the 4D-CT dataset.

- The gross tumor volume (**GTV**) is defined as pathologically confirmed or radiographically suspicious (>1cm in diameter, and/or PET avid) tumor and lymph nodes that the radiation oncologist wishes to treat for palliation. **It is not required to treat all intrathoracic disease, and this is left to the judgment of the radiation oncologist.** Lung windows will be used to contour the primary tumor and mediastinal windows for nodal disease.
- Motion management: given that the standard approach in this patient population does not include explicit motion management, options are as follows:

- Option 1: Combine GTVs from different phases of the respiratory cycle (i.e. end exhalation and inhalation) to form an internal GTV (**iGTV**).
- Option 2: Contour GTV on the untagged average or maximum intensity projection to form the iGTV.
- The use of an expansion for microscopic disease is optional and should reflect standard institutional practices. If used, it should be no more than 5 mm and may be adjusted for natural boundaries for spread. If a microscopic expansion is not used, the iGTV can be expanded by 5-8 mm to form PTV. If the microscopic expansion is used, then a further 5 mm expansion to PTV is allowed.

- An esophageal sparing PTV (**ES-PTV**) will be created by subtracting the esophagus_ring (see below) from PTV.

The following OARs will be contoured:

Right and Left Lung

- contoured on the lung window, excluding trachea and GTV

Lung_evaluation

- both lungs minus GTV

Spinal cord

- based on bony limits of the spinal canal, and extending 10 cm above and below the PTV

Esophagus

- contoured on the mediastinal window from cricoid to the GE junction on the average intensity projection. Should include mucosa, muscular, and adventitial layers.

Esophagus_ring

- a 5 mm isotropic expansion around the esophagus to facilitate sparing in the optimization process

Heart

- contoured from the apex of the heart to the inferior aspect of the origination of the ascending aorta

Prior to randomization, the intended APPA fields from Arm 1 will be placed on the planning CT, and that plan will be signed by the radiation oncologist. Those fields will be used as the basis for safe toxicity parameters for Arm 2. If the patient is randomized to Arm 1, the signed fields are to be treated without further modification.

6.1 Standard Arm (Arm 1)

RT for patients in the standard arm should adhere to the principles of palliative RT, with goals of alleviating symptoms or preventing potential complications. An APPA parallel-opposed pair (POP) beam arrangement is favored wherever possible. POP fields will be placed to encompass PTV (without regard for ES-PTV). Multileaf collimator blocks may be placed to protect OARs (i.e. lung) at the discretion of the treating physician.

Dosimetric parameters for Arm 1 are as follows:

- **The field edge should be >5 mm from the edge of PTV.** If the oncologist feels that the resulting field size is larger than required, then smaller target volumes should be defined. It is not necessary to contour areas of disease that are not being treated.
- The maximum effective field size is recommended to be limited to **225 cm^2** but is at the discretion of the treating radiation Oncologist. The maximum hot spot is limited to no more than a 2 cm^3 contiguous volume receiving 115% or more of the prescribed dose.

The treating physician, dosimetrist or physicist will record the following parameters from the APPA fields:

- length of esophagus in the field (defined as the length of esophagus receiving >50% of the prescription dose at any point along its circumference). This is required to be at least 5 cm for enrollment.
- maximum and mean esophageal dose
- maximum cord dose
- PTV D95 (the dose to the hottest 95% of the PTV).
- Lung eval V16 (if 30 Gy in 10 fractions used) or V13 (if 20 Gy in 5 fractions used).
  - The V16 and V13 are used to approximate the V20 used for conventional fractionations. BED values are as follows:
    - When V20 is used with a prescription dose of 60 Gy in 30

fractions, the corresponding BED, for the 20 Gy in 30 fractions received by lung eval, is 24.4 Gy_3

- When V16 is used with a prescription dose of 30 Gy in 10 fractions, the corresponding BED, for the 16 Gy in 10 fractions received by lung eval, is 24.5 Gy_3
- When V13 is used with a prescription dose of 20 Gy in 5 fractions, the corresponding BED, for the 13 Gy in 5 fractions received by lung eval, is 24.2 Gy_3

6.2 Experimental Arm (Arm 2)

The GTVs and PTVs used for planning in Arm 2 are those that have been defined in Arm 1 for that particular patient. Patients randomized to Arm 2 will undergo IMRT planning. Treatment can be delivered using static beams (either 3D-conformal radiotherapy, or intensity-modulated) or rotational therapy (volumetric modulated arc therapy, or tomotherapy). Priority will be placed on generating clinically acceptable plans while minimizing complexity, planning time and treatment time.

ES-PTV will be optimized and the esophagus will take priority over PTV in the optimization process. In most cases, ES-PTV will need to be compromised in order to achieve esophageal constraints.

For plans in Arm 2, dose constraints are as follows:

- The maximum dose within the body is limited to no more than 2 cm^3 receiving 115% or more of the prescription dose.
- The allowable dose to "lung_eval" is based on the values from the fields placed for that particular patient. The V16 value (for 30 Gy in 10 fractions) or the V13 value (for 20 Gy in 5 fractions) may not exceed the value recorded from the APPA fields by more than 3%. For example:
    - if a patient has a V16 value of 30% with the APPA fields (prescription dose 30 Gy in 10 fractions), the V16 value with the IMRT plan may not exceed 33%.
    - if a patient has a V13 value of 30% with APPA fields (prescription dose 20 Gy in 5 fractions), the V13 value with the IMRT plan may not exceed 33%
    - For all patients, the V5 should be less than 60%.
- The dose to the esophagus should be as low as achievable. Underdosage of the PTV is allowed and will often be required. The following approach should be used:
    - **Step 1: A plan is designed constraining the maximum dose to the esophagus to 80% of the prescription dose** (i.e. 24 Gy if using 30 Gy in 10 arm and 16 Gy if using 20 Gy in 5 fractions). This constraint is met regardless of GTV and PTV coverage within the Esophagus_ring. In some cases, if there is GTV within the Esophagus_ring, small areas of that GTV may receive as little as 80% of the prescription dose.
        - 95% of the ES-PTV outside of the Esophagus_ring should still

receive at least the same D95 as in Arm 1. Better PTV coverage is allowed as long as lung constraints are met.

  - **Notes:** suggested optimization parameters will be provided. The maximum esophageal dose is defined as the dose to the hottest 0.1 cc.

- **Step 2. Once this is achieved, the dose to the esophagus should be further reduced if possible**, while ensuring that all GTV within the Esophagus_ring receives at least 80% of the prescription dose, and 95% of all ES-PTV outside the Esophagus_ring receives at least 95% of the prescription dose.

<u>6.2.1 Example Plans for Arm 2</u>

The plans on the following page demonstrate the esophageal sparing technique for two cases prescribed 30 Gy in 10 fractions, one where the GTV is in close proximity to the esophagus (left) and one not in close proximity (right). Structures are: GTV (blue), PTV (red), esophagus (green). The same color scheme applies for the DVHs, with the "lung evaluation" structure in light blue, the dotted lines representing the IMRT plan, and the solid line representing the APPA plan.

Notes:

i. The fields in Arm 1 should represent the fields that would be used in standard clinical practice, and dosimetric coverage of GTV and PTV is not protocol-specified.
ii. In the plan at left, esophageal sparing stops once <0.1 cc of esophagus is receiving ≤80% of the prescription dose. This ensures that the GTV adjacent to the

esophagus receives at least 80% of the prescription dose. In the plan at right, more esophageal sparing can be achieved.

iii. For this prescription, V16 is the parameter of interest for the lung evaluation structure. The V16 in the IMRT plan cannot be >3% more than in the APPA plan, and the V5 must remain below 60%

iv. In some cases, PTV coverage is better with IMRT than with the standard clinical fields. This is allowed, but not required.

v. In a situation where the esophagus is abutting the GTV, GTV underdosage (<80% of the prescription dose) is permissible, provided that this volume is <1cc.

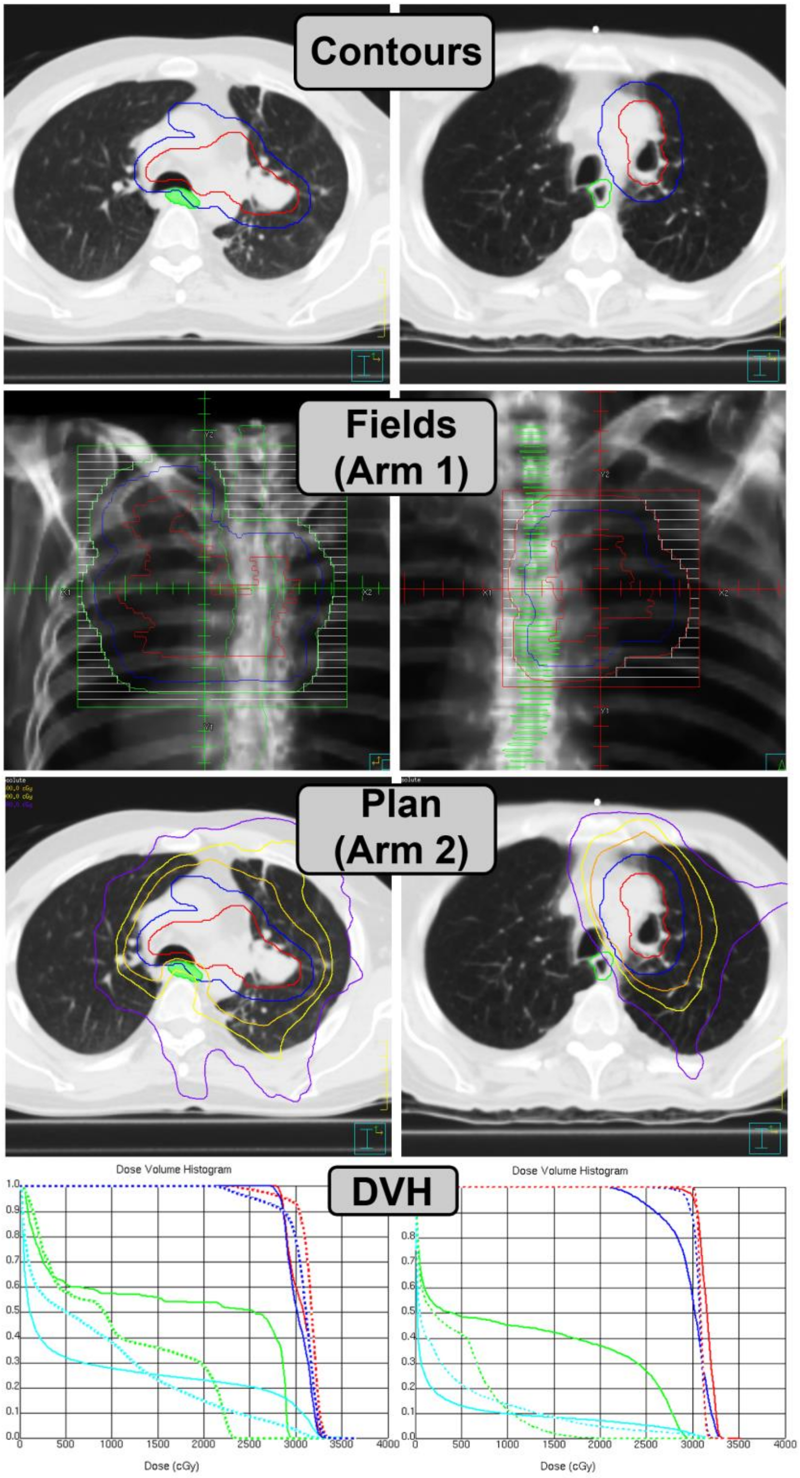

Figure 2: Esophageal Sparing for two cases prescribed 30 Gy in 10 fractions, one where the GTV is in close proximity to the esophagus (left) and one not in close proximity (right). Legend: GTV (blue), PTV (red), esophagus (green). The same color scheme applies for the DVHs, with the "lung evaluation" structure in light blue, the dotted lines representing the IMRT plan, and the solid line representing the APPA plan.

6.3 Image guidance

Daily image guidance is required in both arms, and may be in the form of MV, kV or CBCT imaging. In arm 2, daily CBCT is preferred.

6.4 Central Collection of Imaging Data

Imaging data will be centrally collected through the Quantitative Imaging for Personalized Cancer Medicine (QIPCM) platform (qipcm.technainstitute.com), which provides centralized storage and data analysis tools for medical imaging, and is compliant with national and international privacy regulations. Scans collected by QIPCM will include the planning scan, POP and ES-IMRT radiation dose distributions, and baseline/follow-up CTs of the thorax. Note the follow-up CTs are optional, but those performed will be collected through QIPCM. Participating sites will be provided separate instructions regarding the uploading procedure.

6.5 Registration procedure and Data Collection:

Registration/Randomization Procedure

1. Call the coordinating centre at the Cancer Clinical Research Unit (519-685-8618 Ext. 58623) of London Health Sciences Centre (LHSC) to notify of new randomization and ask for the next patient ID number.
2. Complete the Enrollment form in REDCap prior to randomization. Signed letter of information to be completed and uploaded on to the REDCap database.

3. Notify the coordinating centre that you have completed the enrollment form and uploaded the required documents.

4. If the patient is eligible the coordinating centre will confirm via email and provide the randomization arm.

Data Collection

- For radiation plans that require feedback (e.g. test plans before activation), centres must notify the coordinating centre by phone or email once test plans are uploaded to QIPCM. The coordinating centre will then notify the PIs that the plans are ready to review. After review, the PI will report the acceptability of the plans to the coordinating centre, who will then inform the submitting site.
- All SAEs will be reported using a paper form faxed to the coordinating centre, followed by a call to the coordinating centre immediately after faxing.
- All other data will be collected using REDCap, a web based electronic database.

7.0 ADVERSE EVENTS

7.1 Definitions

Adverse Event (AE) or reaction is any unfavorable and unintended sign (including an abnormal laboratory finding), symptom, or disease temporally associated with the use of a medical treatment or procedure that may or may not be considered related to the medical treatment or procedure.

Serious Adverse Event (SAE) or reaction as defined in the ICH Guideline: Clinical Safety Data Management: Definitions and Standards for Expedited Reporting, E2A Section IIB includes any untoward medical occurrence that at any dose:

- Results in death
- Is life-threatening (refers to an event in which the patient was at risk of death at the time of the event; it does not refer to an event which hypothetically might have caused death if it were more severe.)
- Results in persistent or significant disability/incapacity
- Requires in-patient hospitalization or prolongation of existing hospitalization
- Is a congenital anomaly/birth defect

Important medical events that may not be immediately life-threatening or result in death or hospitalization may be considered a serious adverse event, when, based upon medical and scientific judgment, they may jeopardize the patient or may require intervention to prevent one of the other outcomes listed in the definition above.

Unexpected adverse reaction is one that the nature and severity is not consistent with the applicable product information (e.g., Investigator's Brochure or Product Monograph, described in the REB/IRB approved research protocol or informed consent document), or occurs with more than expected frequency.

7.2 Causality (attribution)

An adverse event or reaction is considered related to the research intervention if there is a reasonable possibility that the reaction or event may have been caused by the research intervention (i.e. a causal relationship between the reaction and the research intervention cannot be ruled out by the investigator(s)).

The relationship of an AE to the study treatment (causality) will be described using the following definitions:

Unrelated

- Any adverse event for which there is evidence that an alternative etiology exists or for which no timely relationship exists to the administration of the study treatment and the adverse event does not follow any previously documented pattern. The adverse event, after careful consideration by the investigator, is clearly and incontrovertibly due to causes other than the intervention.

Unlikely

- Any adverse event for which the time relationship between the study treatment and the event suggests that a causal relationship is unlikely and/or the event is more likely due to the subject's clinical condition or other therapies concomitantly administered to the subject.

Possible

- Any adverse event occurring in a timely manner after the administration of the study treatment that follows a known pattern to the intervention and for which no other explanation is known. The adverse event, after careful consideration by the investigator, is considered to be unlikely related but cannot be ruled out with certainty.

Probable

- Any adverse event occurring in a timely manner after the administration of the study treatment that follows a known pattern to the intervention and for which no other explanation is known. The adverse event, after careful consideration by the investigator, is believed with a high degree of certainty to be related to the intervention.

Definitely Related

- Any adverse event occurring within a timely manner after administration of the study treatment that is a known sequela of the intervention and follows a previously documented pattern but for which no other explanation is known. The adverse event is believed by the investigator to be incontrovertibly related to the intervention.

7.3 Severity

The severity of adverse events will be evaluated using the Common Terminology Criteria for Adverse Events (CTCAE) v4.0 grading scale (see (http://ctep.cancer.gov).

Grade 1: Mild

Grade 2: Moderate

Grade 3: Severe

Grade 4: Life-threatening or disabling

Grade 5: Death

Note: The term “severe” is a measure of intensity: thus a severe adverse event is not necessarily serious. For example, nausea of several hours’ duration may be rated as severe, but may not be clinically serious.

7.4 Immediately Reportable Adverse Events

Any grade 4 or 5 adverse reaction that is definitely, probably, or possibly the result of protocol treatment must be verbally reported to the Principal Investigator and the coordinating centre within 24 hours of discovery, and to the approving research ethics board (REB) as per their reporting guidelines.

Local and non-local SAEs will be reported to the applicable REB as per their reporting guidelines. All serious, unexpected adverse events or reactions regardless of causality

will be reported within 7 days of discovery to the pertinent REB.

NOTE: conditions that are NOT related to protocol treatment are not considered a SAE in this protocol: e.g. hospitalizations for routine procedures, disease progression, toxicity from chemotherapy delivered >2 weeks after radiotherapy.

The Principal Investigator should also comply with the applicable regulatory requirement(s) related to the reporting of unexpected serious adverse drug reactions to the regulatory authority(ies).

8.0 SUBJECT DISCONTUATION/WITHDRAWAL

Subjects may voluntarily discontinue participation in the study at any time. If a subject is removed from the study, the clinical and laboratory evaluations that would have been performed at the end of the study should be obtained. If a subject is removed because of an adverse event, they should remain under medical observation as long as deemed appropriate by the treating physician.

9.0 FOLLOW-UP EVALUATION AND ASSESSMENT OF EFFICACY

Patients will be seen at 2 weeks post radiotherapy. This assessment will be prior to the potential start of any systemic treatment. Further follow-up will be at 2 weeks, 4 weeks, 8 weeks, 3, 6 and 12 months post-radiotherapy. At each visit, the oncologist will conduct a history and physical examination, and CTC-AE toxicities recorded. The FACT-G, its ECS

and LCS subscales, as well as the EQ-5D quality of life questionnaire are to be completed at each visit. (Appendix 2). Please note that if either the treating physician or patient prefer, visits at 4 and 8 weeks may be substituted with a telephone interview (by the research coordinator or physician) to complete quality of life questionnaires and toxicity data. CT chest, is suggested but optional at 3, 6, 9 and 12 months, with additional surveillance imaging and/or laboratory investigations at the discretion of the treating oncologists. Further treatment, (e.g. chemotherapy, targeted therapy, immunotherapy) is at the discretion of the treating oncologists.

9.1 Measurement of Outcomes

9.1.1. Time-to-event outcomes

Overall survival will be measured as time from randomization to death from any cause.

10.0 STATISTICS AND SAMPLE SIZE CALCULATIONS

10.1 Randomization

Patients will be randomized in a 1:1 ratio between Arm 1 and Arm 2. The sample size allows for one stratification factor at randomization: the prescribed RT dose. Randomization will occur in permuted blocks, with the block size known only by the statistician.

A randomized design is indicated for the following reasons:

1. Historically, esophagitis following RT for lung cancer has been measured mostly

through CTCAE, and such data may not provide an appropriate control due to various sources of biases from patient selection, technical RT factors and other comparisons. Thus randomization will provide a more appropriate control group to serve for the experimental arm, particularly when using the FACT-G and cancer subscales.

2. The sample size calculated allows for adequate power to detect a clinically meaningful change in esophageal quality of life

<u>10.3 Stratification</u>

The sample size calculated allows for one stratification factor at randomization, which will be the prescribed dose (20 Gy/5# vs. 30 Gy/10#).

<u>10.4 Sample size calculation</u>

Regarding the primary endpoint, a change in ECS score of 2 to 3 points is considered clinically relevant. We will assume there will be a moderately large effect size (0.65), and a non-responder rate of 15% (5). Using a two-sided, independent-sample t-test with an alpha level of 0.05 and power of 80%, to detect a clinically relevant ECS change in the experimental arm, 90 patients will be required (45 in each arm).

We anticipate that 1-2 patients could be accrued each month at each participating institution, yielding approximately 30 patients per year. The study thus projects accrual over 4 years with 12 months of additional follow-up. Accrual for the sample size calculated is feasible in the Canadian multi-institutional context.

10.5 Analysis plan

Patients will be analyzed according to the intention-to-treat principle (analyze in the groups to which they are assigned).

Primary Endpoint

Esophageal quality of life will be calculated by tabulating FACT-E scores at 2 weeks, with differences between groups tested using the Student's t-test.

Secondary Endpoints

Additional QoL endpoints will be analyzed by compiling relevant EQ-5D values, FACT subscale and trial outcome index (TOI) scores. Treatment arm differences will be analyzed using linear mixed-effects models. Survival will be calculated Kaplan-Meier method with differences compared using the stratified log-rank test. Differences in rates of grade 2 or higher toxicity between groups will be tested using the Fisher's Exact test. Differences in the number of cycles of further systemic therapy will be tested using the student's t-test. EQ-5D measurements will be converted into utilities to inform cost-effectiveness/utility analyses. Finally dosimetric comparison of GTV, PTV, as well as standard pulmonary and esophageal metrics will be compared using the student's t-test.

10.6 Data Safety Monitoring Committee

The DSMC will be independent and consist of a statistician and two content experts uninvolved in the trial. The DSMC will meet every six months after study initiation to review toxicity outcomes. If any grade 3-5 toxicity is reported, the DSMC will review cases notes to determine if such toxicity is related to treatment. If the DSMC deems that toxicity rates are excessive (>25% grade 3 toxicity or >5% grade 4/5 toxicity) then the DSMC can, at its discretion, recommend cessation of the trial or dose adjustment. The DSMC may consider removing a dose level if the rate of clinically significant change in ECS (defined as 2 point change from baseline) occurs in <10% of patients in the control arm. If the DSMC decides that review of toxicity data by email (rather than in-person DSMC meetings) is sufficient because of low toxicity rates, this will be acceptable.

Interim analysis: the DSMC will conduct one interim analysis once 30 patients are accrued. The rationale for this analysis is to ensure that trial accrual is proceeding in a timely manner and to terminate the trial if early stopping rules are met. In this analysis, the DSMC will be blinded to the identity of each treatment arm, but ECS data and OS will be presented for each arm. The DSMC will recommend stopping the trial if there is a statistically significant difference with $p<0.001$ using Student's t-test (Haybrittle-Peto stopping rule) in ECS or OS between the two arms. Furthermore, if the measured differences in esophageal QOL are substantially different than estimated in the sample size calculation, the DSMC can recommend increasing or decreasing the target accrual in order to maintain statistical power.

11. ETHICAL CONSIDERATIONS

The Principal Investigator will obtain ethical approval and clinical trial authorization by competent authorities according to local laws and regulations.

11.1 Institutional Review Board (IRB) / Research Ethics Board (REB)

The protocol (and any amendments), the informed consent form, and any other written information to be given to subjects will be reviewed and approved by a properly constituted Institutional Review Board (IRB)/Research Ethics Board (REB), operating in accordance with the current federal regulations (e.g., Canadian Food and Drug Regulations (C.05.001); US Code of Federal Regulations (21CFR part 56)), ICH GCP and local regulatory requirements. A letter to the investigator documenting the date of the approval of the protocol and informed consent form will be obtained from the IRB/REB prior to initiating the study. Any institution opening this study will obtain REB IRB/REB approval prior to local initiation.

11.2 Informed Consent

The written informed consent form (Appendix) will be provided to potential study subjects, and should be approved by the IRB/REB. The investigator is responsible for obtaining written informed consent from each subject, or from the subject’s legally acceptable representative in the situation that the subject is unable to provide informed

consent. This should be completed prior to beginning and study procedures and treatment. The investigator should inform the subject (or legally acceptable representative) of all aspects of the study, including risks and benefits involved. Ample time should be allowed for questions prior to deciding about participating, and participation is entirely voluntary, and subjects are free to refuse entry into the study, or withdraw at any time for any reason.

The informed consent must be signed and dated by the subject, or legally acceptable representative, and by the person who conducted the informed consent discussion. A copy of this signed and dated form should be given to the subject or the legally acceptable representative. The process of obtaining informed consent should also be documented in the patient source documents.

11.3 Confidentiality of Subject Records

The names and personal information of study participants will be held in strict confidence. All study records (CRFS, safety reports, correspondence, etc.) will only identify the subject by initials and the assigned study identification number. The investigator will maintain a confidential subject identification list (Master List) during the course of the study. Access to confidential information (i.e., source documents and patient records) is only permitted for direct subject management and for those involved in monitoring the conduct of the study (i.e., Sponsors, CRO's, representatives of the IRB/REB, and regulatory agencies). The subject's name will not be used in any public

report of the study.

12.0 AUTHORSHIP

Upon completion of this project, the results will be published in a peer-reviewed journal and presented at one or more conferences. Authorship on such publications will be decided by the study steering committee, and will be commensurate with the relative accrual of each center and the amount of individual contribution, including study design, patient accrual, and data analysis.

13.0 FINANCIAL SUPPORT

This study is partially funded by a grant from the Ontario Institute of Cancer Research through funding provided by the Government of Ontario, the London Health Sciences Centre, and a philanthropic donation. The granting agencies are not directly involved in data collection or analysis.

REFERENCES

[1] Fairchild A, Harris K, Barnes E, et al. Palliative thoracic radiotherapy for lung cancer: A systematic review. *J Clin Oncol* 2008;26:4001-4011.

[2] Bradley JD, Paulus R, Komaki R, et al. Standard-dose versus high-dose conformal radiotherapy with concurrent and consolidation carboplatin plus paclitaxel with or without cetuximab for patients with stage iiia or iiib non-small-cell lung cancer (rtog 0617): A randomised, two-by-two factorial phase 3 study. *Lancet Oncol* 2015;16:187-199.

[3] Kelsey CR, Das S, Gu L, et al. Phase 1 dose escalation study of accelerated radiation therapy with concurrent chemotherapy for locally advanced lung cancer. *Int J Radiat Oncol Biol Phys* 2015;93:997-1004.

[4] Al-Halabi H, Paetzold P, Sharp GC, et al. A contralateral esophagus-sparing technique to limit severe esophagitis associated with concurrent high-dose radiation and chemotherapy in patients with thoracic malignancies. *Int J Radiat Oncol Biol Phys* 2015;92:803-810.

[5] Cella D, Herbst RS, Lynch TJ, et al. Clinically meaningful improvement in symptoms and quality of life for patients with non-small-cell lung cancer receiving gefitinib in a randomized controlled trial. *J Clin Oncol* 2005;23:2946-2954.

APPENDIX 1: ELIGIBILITY CHECKLIST

Eligibility – all answers must be YES, unless specified

Has AJCC 7th edition stage IV NSCLC or stage III not eligible for curative intent treatment	**Yes** / No
Intended to receive palliative RT to thorax, to a dose of 30 Gy in 10 or 20 Gy with > 5cm of esophagus in intended treatment field?	**Yes** / No
Willingness and ability to provide informed consent?	**Yes** / No
Age ≥ 18	**Yes** / No
Performance status: ECOG 0-3	**Yes** / No
Prior planned systematic treatment (chemotherapy, immunotherapy, targeted agents) is permissible, provided **NOT** given 2 weeks prior, concurrent with, or within 2 weeks following RT	
Concurrent palliative RT to other metastatic sites permissible (except lung and/or liver)	
Life expectancy > 3 months	**Yes** / No

Ineligibility – all answers must be NO, unless specified

Prior thoracic RT?	Yes/**No**
Serious medical comorbidities, which preclude the delivery of RT?	Yes/**No**
Is patent pregnant or lactating?	Yes/**No**
Inability to attend the full course of RT or planned follow-up/survey response?	Yes/**No**

Appendix 2 – Follow-up schedule

	pre-RT	2 weeks post RT	*4, 8 weeks post RT	Months 3, 6, 12
History and physical,	X	X	Only if clinic visit is booked	X
*Toxicity scoring	X	X	X	X
CT chest	X			Suggested and optional
Bone scan	Recommended if bony symptoms			
CT or MRI brain	Recommended if CNS symptoms			
*FACT-E and FACT-L	X	X	X	X
*EQ-5D	X	X	X	X

* QoL forms and toxicity scoring may also be completed via telephone for all follow up visits (but not preferred) if patient prefers or is unable to attend clinic. For non-English patients, a fluent family member, staff, or professional translator may assist with completion of the questionnaire. The FACT-E and FACT-L are also available in the following languages (contact the coordinating centre or www.facit.org for these):

Chinese (simplified or traditional), Dutch, French, German, Hungarian, Italian, Japanese, Polish, Spanish

Appendix 3 – Letter of Information (to follow)

Appendix 4 – FACT-G, FACT-E, FACT-L, EQ-5D Quality of Life Forms

Below is a list of statements that other people with your illness have said are important. **Please circle or mark one number per line to indicate your response as it applies to the <u>past 7 days</u>.** This questionnaire will only take 5-10 minutes to complete.

	PHYSICAL WELL-BEING	Not at all	A little bit	Some -what	Quite a bit	Very much
GP1	I have a lack of energy	0	1	2	3	4
GP2	I have nausea	0	1	2	3	4
GP3	Because of my physical condition, I have trouble meeting the needs of my family	0	1	2	3	4
GP4	I have pain	0	1	2	3	4
GP5	I am bothered by side effects of treatment	0	1	2	3	4
GP6	I feel ill	0	1	2	3	4
GP7	I am forced to spend time in bed	0	1	2	3	4

	SOCIAL/FAMILY WELL-BEING	Not at all	A little bit	Some -what	Quite a bit	Very much
GS1	I feel close to my friends	0	1	2	3	4
GS2	I get emotional support from my family	0	1	2	3	4
GS3	I get support from my friends	0	1	2	3	4
GS4	My family has accepted my illness	0	1	2	3	4
GS5	I am satisfied with family communication about my illness	0	1	2	3	4
GS6	I feel close to my partner (or the person who is my main support)	0	1	2	3	4
Q1	*Regardless of your current level of sexual activity, please answer the following question. If you prefer not to answer it, please mark this box ☐ and go to the next section.*					
GS7	I am satisfied with my sex life	0	1	2	3	4

Please circle or mark one number per line to indicate your response as it applies to the past 7 days.

EMOTIONAL WELL-BEING

		Not at all	A little bit	Some -what	Quite a bit	Very much
GE1	I feel sad	0	1	2	3	4
GE2	I am satisfied with how I am coping with my illness	0	1	2	3	4
GE3	I am losing hope in the fight against my illness	0	1	2	3	4
GE4	I feel nervous	0	1	2	3	4
GE5	I worry about dying	0	1	2	3	4
GE6	I worry that my condition will get worse	0	1	2	3	4

FUNCTIONAL WELL-BEING

		Not at all	A little bit	Some -what	Quite a bit	Very much
GF1	I am able to work (include work at home)	0	1	2	3	4
GF2	My work (include work at home) is fulfilling	0	1	2	3	4
GF3	I am able to enjoy life	0	1	2	3	4
GF4	I have accepted my illness	0	1	2	3	4
GF5	I am sleeping well	0	1	2	3	4
GF6	I am enjoying the things I usually do for fun	0	1	2	3	4
GF7	I am content with the quality of my life right now	0	1	2	3	4

Please circle or mark one number per line to indicate your response as it applies to the past 7 days.

	ADDITIONAL CONCERNS	Not at all	A little bit	Some -what	Quite a bit	Very much
HN1	I am able to eat the foods that I like	0	1	2	3	4
HN2	My mouth is dry	0	1	2	3	4
HN3	I have trouble breathing	0	1	2	3	4
HN4	My voice has its usual quality and strength	0	1	2	3	4
HN5	I am able to eat as much food as I want	0	1	2	3	4
HN 10	I am able to communicate with others	0	1	2	3	4
HN7	I can swallow naturally and easily	0	1	2	3	4
E1	I have difficulty swallowing solid foods	0	1	2	3	4
E2	I have difficulty swallowing soft or mashed foods	0	1	2	3	4
E3	I have difficulty swallowing liquids	0	1	2	3	4
E4	I have pain in my chest when I swallow	0	1	2	3	4
E5	I choke when I swallow	0	1	2	3	4
E6	I am able to enjoy meals with family or friends	0	1	2	3	4
C6	I have a good appetite	0	1	2	3	4
E7	I wake at night because of coughing	0	1	2	3	4
ACT 11	I have pain in my stomach area	0	1	2	3	4
C2	I am losing weight	0	1	2	3	4

Please circle or mark one number per line to indicate your response as it applies to the past 7 days.

	ADDITIONAL CONCERNS	Not at all	A little bit	Some-what	Quite a bit	Very much
B1	I have been short of breath	0	1	2	3	4
C2	I am losing weight	0	1	2	3	4
L1	My thinking is clear	0	1	2	3	4
L2	I have been coughing	0	1	2	3	4
B5	I am bothered by hair loss	0	1	2	3	4
C6	I have a good appetite	0	1	2	3	4
L3	I feel tightness in my chest	0	1	2	3	4
L4	Breathing is easy for me	0	1	2	3	4
Q3	Have you ever smoked?<br>No___Yes____If yes:					
L5	I regret my smoking	0	1	2	3	4

Under each heading, please tick the ONE box that best describes your health TODAY.

MOBILITY

I have no problems in walking about ❑

I have slight problems in walking about ❑

I have moderate problems in walking about ❑

I have severe problems in walking about ❑

I am unable to walk about ❑

SELF-CARE

I have no problems washing or dressing myself ❑

I have slight problems washing or dressing myself ❑

I have moderate problems washing or dressing myself ❑

I have severe problems washing or dressing myself ❑

I am unable to wash or dress myself ❑

USUAL ACTIVITIES *(e.g. work, study, housework, family or leisure activities)*

I have no problems doing my usual activities ❑

I have slight problems doing my usual activities ❑

I have moderate problems doing my usual activities ❑

I have severe problems doing my usual activities ❑

I am unable to do my usual activities ❑

PAIN / DISCOMFORT

I have no pain or discomfort ❑

I have slight pain or discomfort ❑

I have moderate pain or discomfort ❑

I have severe pain or discomfort ❑

I have extreme pain or discomfort ❑

ANXIETY / DEPRESSION

I am not anxious or depressed ❑

I am slightly anxious or depressed ❑

I am moderately anxious or depressed ❑

I am severely anxious or depressed ❑

I am extremely anxious or depressed ❑

We would like to know how good or bad your health is TODAY.

- This scale is numbered from 0 to 100.
- 100 means the <u>best</u> health you can imagine.
  0 means the <u>worst</u> health you can imagine.
- Mark an X on the scale to indicate how your health is TODAY.
- Now, please write the number you marked on the scale in the box below.

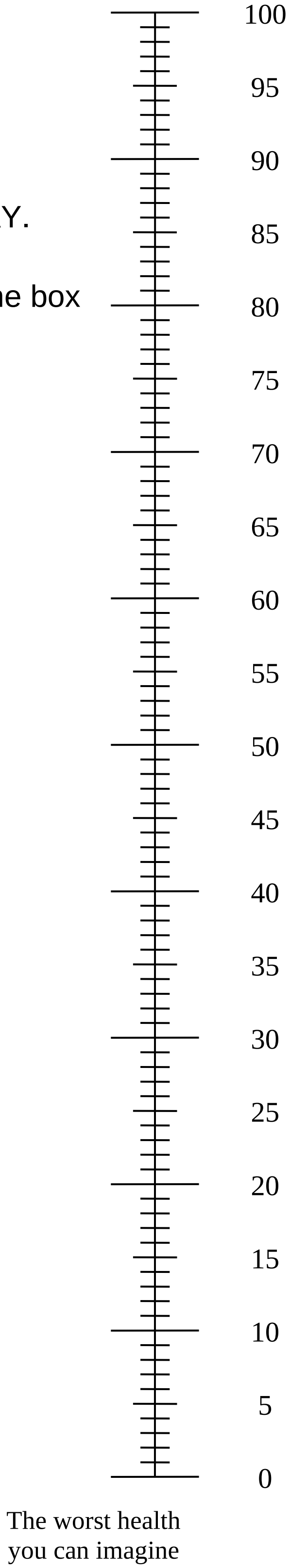

YOUR HEALTH TODAY = [ ]
