# Supplementary material for: Palliative Radiation for Advanced Central Lung Tumors With Intentional Avoidance of the Esophagus (PROACTIVE): A Phase 3 Randomized Clinical Trial
Source: JAMA Oncol. 2022 Feb 24;8(4):1–7. doi: 10.1001/jamaoncol.2021.7664 (PMC8874872; doi:10.1001/jamaoncol.2021.7664)
Supplement: Supplement 2. — eAppendix. Treatment Planning Guidelines for PROACTIVE eAppendix. Treatment Planning Guidelines for PROACTIVE eMethods. Sensitivity Analyses eResults. Sensitivity Analyses eFigure. Changes in Quality-of-Life Scores Over Time by Domain and by Treatment Arm eTable. Subgroup Analyses of FACT-E ECS 2 Weeks Postradiotherapy [file jamaoncol-e217664-s002.pdf]

Supplementary Online Content

Louie AV, Granton PV, Fairchild A, et al. Palliative radiation for advanced central lung tumors with intentional avoidance of the esophagus (PROACTIVE): a phase 3 randomized clinical trial. *JAMA Oncol*. Published online February 24, 2022. doi:10.1001/jamaoncol.2021.7664

eAppendix. Treatment Planning Guidelines for PROACTIVE

eMethods. Sensitivity Analyses

eResults. Sensitivity Analyses

eFigure. Changes in Quality-of-Life Scores Over Time by Domain and by Treatment Arm

eTable. Subgroup Analyses of FACT-E ECS 2 Weeks Postradiotherapy

This supplementary material has been provided by the authors to give readers additional information about their work.

eAppendix. Treatment Planning Guidelines for PROACTIVE

Physics/Dosimtery contact: p.granton@erasmusmc.nl

Study PI: Alexander.Louie@londonhospitals.ca

The experimental arm of PROACTIVE requires IMRT. No specific IMRT technique is specified in the trial protocol. Herein we provide a practical guide for VMAT use but the choice and implementation of IMRT at your centre is discretionary so long as it meets the planning constraints. It is important to understand that this clinical trial prioritizes sparing the esophagus more than achieving complete coverage of the intended treatment region. Plan objectives and constraints for the experimental arm are outlined in table 1. In most cases the planning objects and constraints can be met relatively easily provided that there is some separation between the GTV and the esophagus.

Prior to treatment plan optimization, critical and target structures need to be delineated and optimization structures generated. These structures have been defined and outlined in table 2. The responsible radiation oncologist delineates the GTV and PTV contours; all other structures can be delineated by a Dosimetrist or qualified Medical Radiation Therapist.

Planning constraints for the standard POP arm are not included herein but should be according to institutional guidelines. A frequent objective locally maintained is that the $D100\%_{GTV}$ > 80% of the prescription. Alternatively, it is also possible that coverage of the PTV in the standard arm could be greater than $D_{95\%}$ > 95% Prescription (i.e. 28.5 Gy in the 30/10 arm) for example, if the Norm Point is on the field edge; However these types of plans should be discouraged as it may unfairly challenge the experimental arm to create a ES-IMRT plan with similar coverage and break the spirit of the prescription.

Experimental Arm (30/10 & 20/5)			Comment
Objective	ESPTV	$D95\%_{ES\text{-}IMRT} \geq D95\%_{POP}$	
Constraint	Esophagus	$D_{max(0.1cc)} < 80\%$	Only 0.1 cc of the esophagus can exceed 80% of the prescription.
Constraint	GTV	$D_{min(1cc)} \geq 80\%$	Only 1cc of the GTV can be lower than this value
Constraint	any	$Dmax_{(contiguous\ 2cc)} < 115\%$	The max hot spot is limited to 115% prescription to no more than 2cc contiguous volume
Constraint	Lung_eval	V5 < 60%	Constrain 60% of the lungs to be less than 5 Gy to reduce low dose region in lungs
Constraint	Cord	$Dmax_{(0.1cc)} < 115\%$	i.e. Max 0.1cc dose to the Cord
**Arm. 30/10**			
Constraint	PTV	D99% ≥ 2000 cGy	The minimum dose to 99% of the PTV shall not be less than 2000 cGy
Constraint	Lung_eval	$V16_{ES\text{-}IMRT} \leq (V16_{POP} + 3\%)$	See protocol
**Arm. 20/5**			
Constraint	PTV	D99% ≥ 1350 cGy	The minimum dose to 99% of the PTV shall not be less than 1350 cGy
Constraint	Lung_eval	$V13_{ES\text{-}IMRT} \leq (V13_{POP} + 3\%)$	See protocol

Table 1. A succinct list of the objective and constraints for the experimental arm

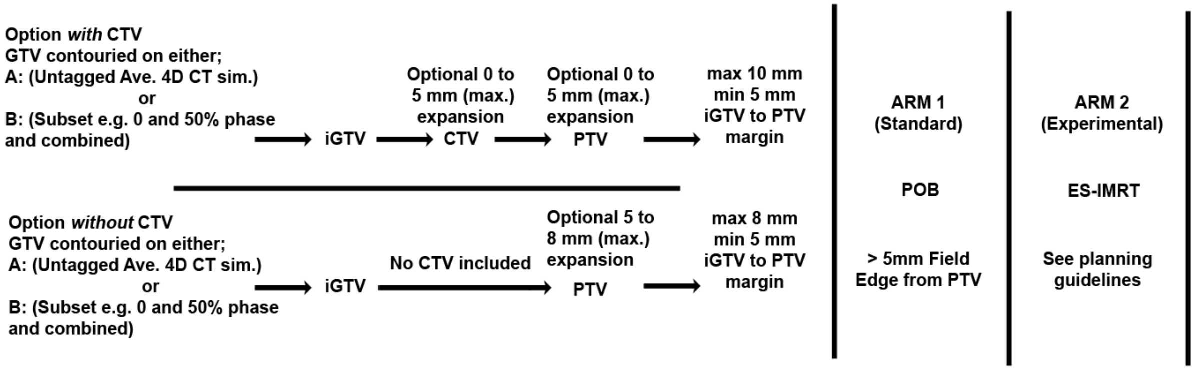

Figure 1. Contouring target delineations for treating radiation oncologist.

	Defined & Required Structures	Defined convention Spelling	Comments
**Target**	GTV	GTV	See protocol & figure 1.
	iGTV	iGTV	See protocol & figure 1.
	CTV	CTV	See protocol & figure 1.
	ITV	ITV	See protocol & figure 1.
	PTV	PTV	See protocol & figure 1.
**OAR structures**	Esophagus	Esophagus	Esophagus from the GE junction to the cricoid
	Esophagus falloff region	Esophagus_ring	0.5 cm ring around Esophagus
	Spinal Cord	Cord	no additional margin
	Heart	Heart	no additional margin
	Left Lung	Lung_L1	no additional margin
	Right Lung	Lung_R1	no additional margin
**Optimizing Structures**	Normal Lung	Lung_eval	Left & Right lung minus the iGTV
	Normal Tissue	Normal_tissue	External body contour minus (PTV + 3 cm margin for rings)
	Compromised GTV	ESGTV	iGTV minus the Esophagus_ring
	Compromised PTV	ESPTV	PTV minus the Esophagus & Esophagus_ring (see figure 2)
	PTV minus ESPTV	PTVminusESPTV	The compromised portion of the PTV
	Esophagus outside PTV	Esophagus_opt	The Esophagus outside the PTV
	PTV ring	PTVRING	PTV – iGTV, structure used to avoid hot spots near the esophagus
	PTV Ring Overlap	PTVRING_Overlap	PTV overlapping with Esophagus_ring
	GTV Ring Overlap	GTVRING_Overlap	iGTV overlapping with Esophagus_ring Min (80 % Prescription)
	Ring 1	Ring_1	1 cm ring surrounding the PTV
	Ring 2	Ring_2	1 cm ring surrounding Ring_1
	Ring 3	Ring_3	1 cm ring surrounding Ring_2

Table 2. Definition of the critical structures and target volumes

Contouring Guidelines for elective VMAT:

Prior to treatment plan optimization it is important to ensure that the contours have been delineated well and do not create any conflicts. In particular, since some elements are delineated separately there maybe be regions of overlap between two critical structures that may require adjustments. For some patients if the gross disease abuts the esophagus, it is very possible that the delineated GTV from the 4-D CT phases may overlap partially with the esophagus that is contoured on the untagged average image. This overlap needs to be resolved for the optimization process to proceed correctly.

It is also important to critically review the esophagus contour and ensure that they are contiguous i.e. there are no jumps between slices particularly near the GTV and that the inferior/superior extent of the esophagus is according to protocol.

Small modifications to the ESPTV are permitted if the structure exhibits features that would make optimization unfeasible, such as sharp spurs (i.e. see Figure 2) below.

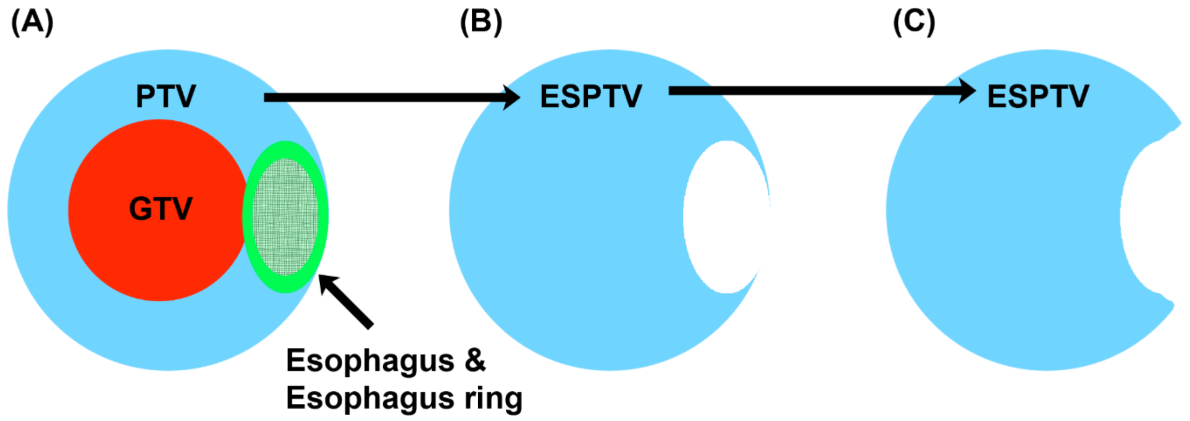

Figure 2. Small modifications to the ESPTV that would be deemed acceptable.

Planning Guidelines:

Assuming a patient has met the trial criteria for inclusion and critical structures have been delineated, it is important to initialize and set-up the optimization problem/treatment plan using good planning practices. Good planning practices suggest the following modifications to the treatment plan occur to achieve the desired outcomes.

1. If using VMAT, ensure that the treatment arc rotates around an isocentre at or near the center of the target volume by creating a new reference point isocentre (if necessary) in the center of the target volume, feel free to round small differences from the set-up isocentre e.g. if you created a new isocentre at 2.4, -0.3, 3.1 cm, please feel free to use 2.5, 0, 3 cm. Beam associated with the plan are attached to this isocentre.

2. Rotate the collimator so that the axis of elongation is perpendicular to the leaf travel so that the multi-leaf collimators have a smaller distance to travel, which will also increase its potential to modulate the field (45 degrees has found to be a common collimator angle).

3. At the desired collimator angle, determine if two arcs are required to cover the target volume. If a single arc can cover the target volume, than optimizing using a single arc should first be tried and two arcs only deployed if better target coverage is required. Two arcs may improve target coverage at the expense of a low dose wash throughout the organs at risk.

4. Enable an isotropic dose-calculation grid of at least 0.3 cm^3 and final segments spaced at minimum of 4 degrees so that a full 360° will create 91 segments.

5. Enable VMAT, set the energy to be 6 MV, dose rate to be 600 MU and constrain the maximum leaf travel motion is set appropriately for your institution (at LHSC this is 0.46 cm/deg).

6. Feel free to make use of jaw tracking if you have it enable and commissioned, otherwise leave jaw tracking off.

7. Use direct aperture optimization to ensure that optimizations are deliverable.

8. Use a sufficient number of optimization iterations and computer an intermediate dose calculation. At LCRP 40 max iterations are used between optimizations and dose convolutions occurs at the 20th iteration.

Description of the optimization process

Once the treatment planning processes has reached the point of initializing the optimization process the objectives and constraints need to be defined. Table 3 and 4 list suggested initial values (from 0 to 100) and parameters to set-up the optimization problem that has result in good final treatment plans. Please use at your discretion and feel free to modify based on your own treatment planning experience.

Structure	Parameter	Value (Gy)	Volume	VMAT Weight
**ESGTV**	Max Dose	3125	-	50
**ESGTV**	Min Dose	3050	-	60
**ESPTV**	Max Dose	3100	-	50
**ESPTV**	Min Dose	3050		60
**ESPTV**	Uniform Dose	3075	-	5
**Esophagus**	Max Dose	2400	-	100
**Esophagus_opt**	Max DVH	200	50	20
**Esophagus_opt**	Max DVH	80	25	50
**Esophagus_opt**	Max DVH	1400	10	60
**PTVRING_Overlap**	Max Dose	2800	-	20
**GTVRING_Overlap**	Min Dose	2350	-	20
**PTVminusESPTV**	Max Dose	2400	-	80
**PTVminusESPTV**	Min Dose	2000	-	100
**PTVRING**	Max Dose	3200	-	20
**PTVRING**	Min Dose	3025	-	20
**Lung_eval**	Max DVH	500	50	40
**Lung_eval**	Max DVH	1600	20	40
**Ring_1**	Max Dose	3000	-	20
**Ring_2**	Max Dose	2400	-	20
**Ring_3**	Max Dose	1800	-	20
**Heart**	Max DVH	2400	10	10
**Heart**	Max Dose	2600	-	30
**Cord**	Max Dose	3050	-	50

Table 3. Initialization of the objective function and optimization process for 30 Gy/10 fractions

Once a first optimization run has occurred, review the residual objective function values in order to determine if some objective can be modified to improve the treatment plan. In general, for each objective parameter an objective value of no more than 10 % of the total represents a good value, and would indicate that the optimizing is not conflicted but working appropriately on the objective parameter.

Once the optimization has stabilized separate structures can be generated to boost the periphery of the target to ensure good coverage. The treatment planner is at his or her discretion as to best to proceed to achieve the treatment goals.

Structure	Parameter	Value (Gy)	Volume	VMAT Weight
ESGTV	Max Dose	2150	-	50
ESGTV	Min Dose	2050	-	60
ESPTV	Max Dose	2130	-	50
ESPTV	Min Dose	2025		60
ESPTV	Uniform Dose	2100	-	5
Esophagus	Max Dose	1600	-	100
Esophagus_opt	Max DVH	150	50	5
Esophagus_opt	Max DVH	650	25	5
Esophagus_opt	Max DVH	950	10	5
GTVPTV_Overlap	Max Dose	1600	-	20
PTVRING	Max Dose	2150	-	20
PTVRING	Min Dose	2025	-	20
PTVminusESPTV	Max Dose	1600	-	80
PTVminusESPTV	Min Dose	1350	-	100
Lung_eval	Max DVH	500	50	40
Lung_eval	Max DVH	1300	20	40
Ring_1	Max Dose	2000	-	20
Ring_2	Max Dose	1600	-	20
Ring_3	Max Dose	1200	-	20
Heart	Max DVH	950	10	10
Heart	Max Dose	1750	-	30
Cord	Max Dose	2300	-	50

Table 4. Initialization of the objective function and optimization process for 20 Gy/5 fractions

eMethods. Sensitivity Analyses

All logistic regression models were stratified by intended dose. To reduce small sample bias, the Firth's Penalized Likelihood method (Firth's method) was applied to logistic regression models with intended dose as a fixed effect (predictor). This approach could not be applied to models with intended dose as a stratification factor given the limitation that these adjustments cannot be applied simultaneously to the same model.

eResults. Sensitivity Analyses

Compared to standard RT arm, significant increases in ES-IMRT arm were observed for planned esophagus length < 10 cm (55.2 ± 7.6 vs. 49.4 ± 7.8; *P*=.02) and stage III disease (54.3 ± 6.7 vs. 46.3 ± 9.7; *P*=.03), however no significant differences were observed for planned esophagus length ≥ 10 cm (*P*=.70), adenocarcinoma (*P*=.06), squamous cell carcinoma (*P*=.17), other histology (*P*=.18), stage IV disease (*P*=.55) or ECOG performance status 0-1 (*P*=.17) or 2-3 (*P*=.36).

None of the interactions between treatment arm and planned esophagus length, histology, stage and ECOG performance status were significant. In a post-hoc subgroup analysis dividing patients by the stratification factor, this benefit was mostly observed in patients receiving 30 Gy (51.1 ± 10.7 vs. 56.4 ± 7.1, *P*=.06), rather than in those receiving 20 Gy (49.7 ± 9.8 vs. 50.9 ± 7.2, *P*=.68). The interaction between treatment arm and intended dose was not significant (*P*=.31), however this study was not powered to detect statistical significance on interaction tests.

The interaction between treatment arm and intended dose was not significant (*P*=.23). As a sensitivity analysis to evaluate the impact of participating institution, multivariable logistic regression analysis was repeated also adjusting for institution. Similar results were observed after adjusting for institution.

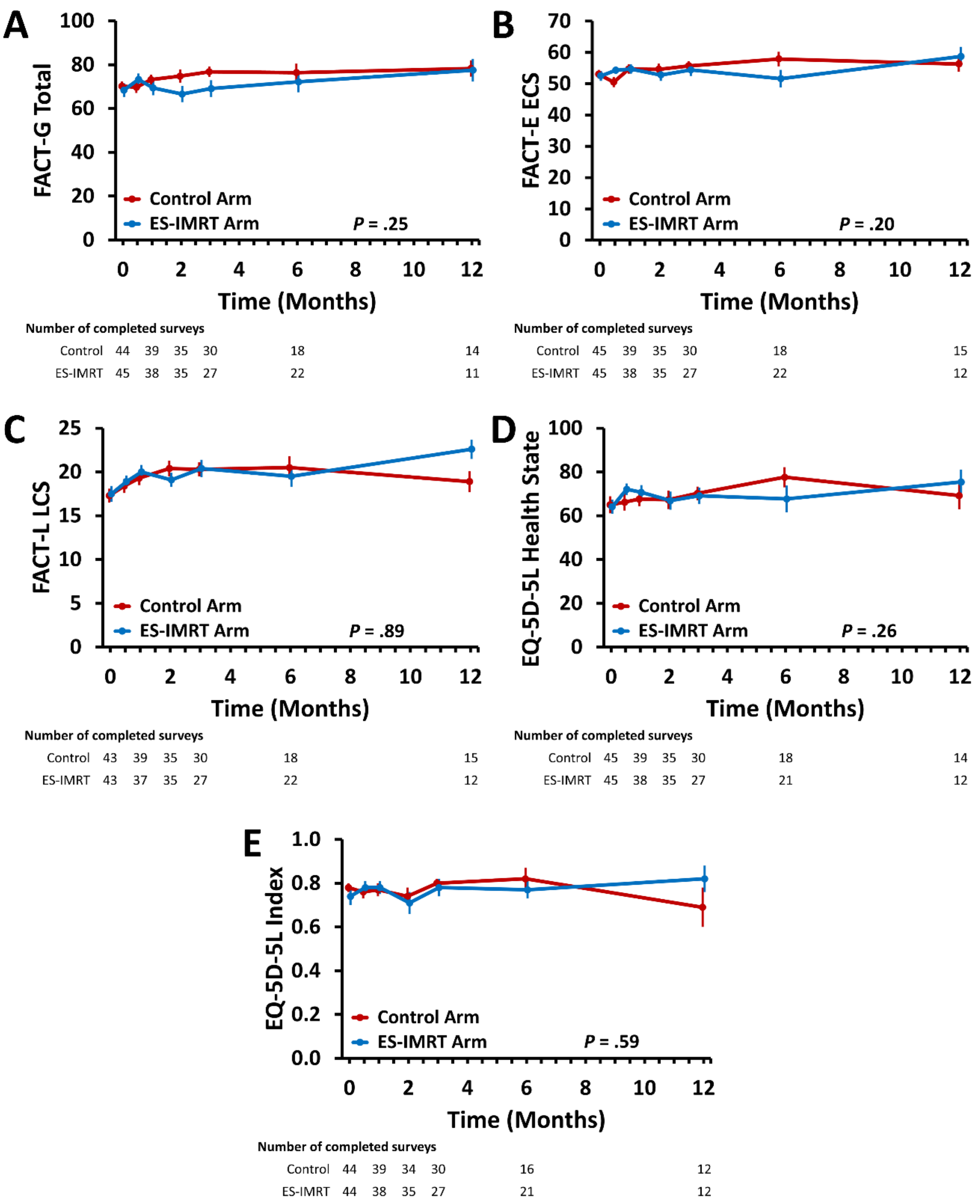

eFigure. Changes in quality-of-life scores over time by domain and by treatment arm. P-values are shown for interaction between treatment arm and time reported from linear mixed models adjusting for time, treatment arm and intended dose as fixed effects and patient number as a random effect. Abbreviations: ES-IMRT – esophageal-sparing intensity-modulated radiotherapy; FACT-G - Functional Assessment of Cancer Therapy: General; FACT-E - Functional Assessment of Cancer Therapy: Esophagus; FACT-L - Functional Assessment of Cancer Therapy: Lung; ECS - esophageal cancer subscale; LCS - lung cancer subscale; EQ-5D-5L - EuroQoL 5-Dimension 5-Level.

eTable. Subgroup analyses of FACT-E ECS 2 weeks postradiotherapy.

Variable	Control Arm (n=45)	ES-IMRT Arm (n=45)	P-value [a]	Interaction P-value [b]
**Planned esophagus length –** mean ± SD				
< 10 cm	49.4 ± 7.8	55.2 ± 7.6	**.02**	.29
≥ 10 cm	51.2 ± 11.6	52.4 ± 7.3	.70	
**Histology** – mean ± SD				
Adenocarcinoma	50.2 ± 10.7	56.4 ± 7.3	.06	.10
Squamous	48.0 ± 11.5	53.5 ± 7.6	.17	
Non-small cell lung cancer / not otherwise specified	53.5 ± 7.9	48.7 ± 6.0	.18	
**Stage** – mean ± SD				
III	46.3 ± 9.7	54.3 ± 6.7	**.03**	.14
IV	52.8 ± 9.9	54.3 ± 7.9	.55	
**ECOG performance status –** mean ± SD				
0 - 1	52.6 ± 7.7	55.5 ± 7.8	.17	.82
2 - 3	46.2 ± 13.3	50.0 ± 5.0	.36	
**Intended dose (post-hoc analysis)** – mean ± SD				
20 Gy in 5 fractions	49.7 ± 9.8	50.9 ± 7.2	.68	.31
30 Gy in 10 fractions	51.1 ± 10.7	56.4 ± 7.1	.06	

[a] Reported from independent-sample t-test comparing treatment arms (“difference”);
[b] Reported from interaction term between treatment arm and variable shown;
Abbreviations: ES-IMRT – esophageal-sparing intensity-modulated radiotherapy; SD – standard deviation; FACT-E – Functional Assessment of Cancer Therapy: Esophagus; ECS – Esophageal cancer subscale; ECOG – Eastern Cooperative Oncology Group; P-values < 0.05 shown as BOLD.
