# Supplementary material for: Palliative Radiation for Advanced Central Lung Tumors With Intentional Avoidance of the Esophagus (PROACTIVE): A Phase 3 Randomized Clinical Trial
Source: JAMA Oncol. 2022 Feb 24;8(4):1–7. doi: 10.1001/jamaoncol.2021.7664 (PMC8874872; doi:10.1001/jamaoncol.2021.7664)
Supplement: Supplement 3. — Data Sharing Statement [file jamaoncol-e217664-s003.pdf]

Data Sharing Statement

Louie. Palliative Radiation for Advanced Central Lung Tumors With Intentional Avoidance of the Esophagus (PROACTIVE). *JAMA Oncol*. Published February 24, 2022. doi:10.1001/jamaoncol.2021.7664

Data
Data available: No
